# A bias-minimising measure of the influence of head orientation on perceived gaze direction

**DOI:** 10.1038/srep41685

**Published:** 2017-01-31

**Authors:** Tarryn Balsdon, Colin W. G. Clifford

**Affiliations:** 1 School of Psychology, UNSW Australia, Sydney, NSW, Australia

## Abstract

The orientation of the head is an important cue for gaze direction, and its role has been explained in a *dual route* model. The model incorporates both an *attractive* and a *repulsive* effect of head orientation, which act to support accurate gaze perception across large changes in natural stimuli. However, in all previous studies of which we are aware, measurements of the influence of head orientation on perceived gaze direction were obtained using a single-interval methodology, which may have been affected by response bias. Here we compare the single-interval methodology with a two-interval (bias-minimising) design. We find that although measures obtained using the two-interval design showed a stronger attractive effect of head orientation than previous studies, the influence of head orientation on perceived gaze direction still represents a genuine perceptual effect. Measurements obtained using the two-interval design were also shown to be more stable across sessions one week apart. These findings suggest the two-interval design should be used in future experiments, especially if comparing groups who may systematically differ in their biases, such as patients with schizophrenia or autism.

Accurate perception of gaze direction is important as a means of unspoken communication and is integral to understanding others’ beliefs, intentions, and desires. Humans have been shown to be very good at inferring where someone is looking^1^,^2^, and it has even been suggested that the human eye has evolved to promote this ability, with greater accentuation of the whites of the sclera against the dark pupil, in comparison to other primates^3^,^4^. This feature enables one to more easily judge the direction of another’s gaze from the relative position of the dark pupil against the visible sclera.

However, it is not only information concerning the pupil and the sclera that is incorporated in perceiving the direction of another’s gaze. Evidence suggests that a multitude of other information, exterior to the eye region, is used to optimise the perception of gaze direction across large changes in natural stimuli both for accuracy and social value, such as the emotional expression^5^, contextual objects^6^, and prior expectations^7^,^8^,^9^,^10^.

Another important cue in perceiving the direction of another’s gaze is the rotation of the head. In one respect, the direction of the head acts as a coarse scale spatial cue to the direction of gaze; observers are quicker to judge the direction of gaze when the eyes and the head are oriented in the same direction^11^,^12^,^13^,^14^ and direct gaze is reported over a wider range of gaze deviations when a stimulus with a direct head is presented in the periphery^14^. In another respect, as the head rotates, the visible portion of the eyes change, altering the spatial distribution of light and dark regions projected to the observer. If the head rotates to the right there is an increase in the relative amount of visible sclera to the right of the pupil, just as when the pupil direction alone is shifted to the left. Thus, in calculating the position of the pupil within the visible sclera, one must also account for what part of the sclera is visible. The rotation of the head therefore changes two important cues in perceiving gaze direction: the coarse spatial scale cue and the eye region information.

A *dual route* model has been proposed to explain how head orientation might bias the perception of gaze direction^15^,^16^. Under this model, the rotation of the head has a direct attractive effect on the perceived gaze direction, pulling the perceived direction of gaze towards the direction of head orientation. This tends to counteract an indirect repulsive effect on the eye region information in accordance with the relative deviation of the pupil within the visible part of the sclera. Jointly, these two effects work together to keep the accuracy with which we perceive gaze direction relatively constant, despite large changes in natural stimuli. The model is also able to explain peculiarities in the perception of gaze direction such as in the Wollaston effect, wherein the perceived direction of gaze changes when the same eyes are placed within heads of differing orientations^17^.

The dual route model helps to separate how eye deviation and information from the head might be differently weighted in perceiving gaze direction. If the orientation of the head causes gaze direction to be perceived more in the opposite direction of the head (than is veridically presented), this is an increased repulsive effect, which can be described as a negative weighting of the total effect of head orientation (from Fig. 1). Whereas, if the orientation of the head causes gaze direction to be perceived more in the same direction as the orientation of the head, the repulsive effect is decreased, showing a positive weighting of head information.

This model can be used to distinguish between effects localised to the eye region and effects from integrating information from the whole head. As such, it is potentially important for interpreting the abnormal gaze perception demonstrated in certain clinical populations, such as in people with autism spectrum disorder (ASD)^18^,^19^,^20^, schizophrenia^21^,^22^,^23^,^24^, and social anxiety disorder^25^,^26^,^27^. For example, Webster and Potter, 2008, found that detecting gaze direction develops slowly in children with ASD, with younger children significantly poorer at judging which of four poles the experimenter was looking at, whilst the experimenter’s head was always directed towards them. Given the suggestion that ASD might be characterised by a local (as opposed to global) information processing style^28^ it could be questioned whether some difference in the weighting of head orientation information in children with ASD could account for the difference in sensitivity to gaze direction.

However, in order to use a methodology to compare clinical populations to control groups one must ensure that any differences are the result of the measured effect, and not some artefact produced by the experimental design. Of particular consideration is the problem of response bias. In the experimental design employed by Otsuka *et al*.^15^,^16^ and other previous studies, participants were shown a single stimulus and asked to respond as to whether they thought gaze was directed to the left, right, or directly towards them. Given that the difference in perceived gaze direction was also accompanied by a noticeable difference in head rotation, the difference may have been exaggerated by a simple tendency to respond a certain way when presented with a head oriented in a certain direction, that is, to have a response bias. Because observers are making a left-right judgement on a stimulus within a left-right context, it is unclear whether observers genuinely perceived the same gaze deviation differently in different head orientation contexts, or if they simply had a tendency to respond in the opposite direction of head orientation. For example, when they were unsure of the gaze direction they might incorporate the head orientation into a default response. The distinction here is between a genuine perceptual effect, where the direction of gaze actually appears different with different head orientations, and a contribution of response bias, where the direction is reported differently with different head orientations, but actually appears the same.

This is particularly important when considering patient populations, as they may differ in their biases. For instance, patients with schizophrenia have been shown to have increased response biases in perceptual and memory tasks^29^,^30^,^31^. Were a task employed that is vulnerable to biases, any difference found between patients and controls could be the result of a systematic difference in bias, as opposed to a genuine difference in the perceptual effect being measured.

The problem of response bias is not limited to gaze perception, as it applies in any case where an observer is making a judgement in the presence of a context that varies in the same manner as the judgement itself; in this case a left-right judgement in the presence of a left-right context. A similar problem has been explored in the tilt illusion, where the perceived orientation of a centre grating is influenced by the orientation of a surround grating^32^. Patten and Clifford^32^ showed that a two-interval, two-alternative forced choice task could be employed to measure the influence of the surround grating orientation on the perceived orientation of the centre grating in a manner free from this particular response bias. In fact, this approach can be meaningfully applied in any case where the measured perception of one stimulus is altered by the presence of another, such as in aftereffects and adaptation^33^,^34^,^35^.

In the two-alternative forced choice task, the observer is presented with two stimuli (in separate spatial or temporal intervals), and asked to respond as to which stimulus was a ‘target’. For example, they are presented with two faces and asked which was looking more directly at them. The two-interval task (if counterbalanced correctly) disconnects the response from the stimulus type – were a participant to have a tendency to choose the first interval more than the second, by default, this would not correspond to choosing a particular stimulus more frequently. Whereas, in the single interval design, where the observer is presented with a single face and asked to decide whether it was looking left or right, the tendency to choose one response more frequently would correspond to a bias for a particular stimulus type, for example, suggesting more faces have leftward gaze, by default. The two interval design, however, does not remove perceptual bias; if the orientation of the head genuinely effects the perceived direction of gaze, then presenting two faces with opposing head orientations will not alter this, and there will still be a measureable difference in perceived gaze direction.

The purpose of this experiment is first, to objectively test whether there is any contribution of response bias to previous measures of head weighting on perceived gaze direction, and second, to demonstrate a bias-minimising methodology for measuring the weighting of head information on perceived gaze direction that can be employed in clinical populations. To do so we will compare measures of the influence of head orientation on perceived gaze direction between single-interval and two-interval task designs. It is expected that if the influence of head orientation on perceived gaze direction is not affected by response bias, then the measures across the two tasks will be similar. We will also test the test-retest reliability of each task, to examine how constant these measures are over time.

## Methods

### Participants

A total of 64 observers participated in this experiment; 49 participants were recruited from the university undergraduate research participation scheme and were given course credit for their participation, and 15 experienced observers also participated. All participants had normal or corrected to normal vision. After the initial examination of the data, 14 participants met exclusion criteria (as described in the Analysis section below) and were excluded, leaving 50 participants in the analysis. All participants provided informed consent and ethical approval for this study was granted by the UNSW Human Research ethics committee, all methods were performed in accordance with the Declaration of Helsinki.

### Apparatus

An 18 inch CRT monitor, with a background luminance of 33.2 cd/m^2^ and resolution 1024 × 768, with a refresh rate of 85 Hz, was used for presenting stimuli. Throughout the experiment, participants sat 57 cm from the screen, with their chin on a chin rest.

### Stimuli

Stimuli were four grey scale faces (two male and two female), with cropped hair and neutral expressions, created with Daz software (http://www.daz3d.com/). Two versions of each face were created, one rotated 15*°* to the right and the other 15*°* to the left. To control for any effect of stimulus asymmetry, on half the trials, a rightward rotated face was presented by vertically flipping the leftward rotated face and similarly, a leftward rotated face was presented by vertically flipping a rightward rotated face. To control eye deviation, the eyes were removed from each face using Gimp software and were replaced with realistic counterparts using a Matlab script that allowed for the eyes to be moved according to precise angular coordinates. Face stimuli were each fit in a box of approximately 400 × 400 pixels in size, subtending approximately 14 × 14 degrees of visual angle. Artificial faces were used to accurately control the deviation of the head and eyes. It is unclear to what extent the results might be affected if real faces were employed; previous experiments have found observers to display superior discrimination of gaze direction when a real person is used^1^, whilst others have shown that the cone of direct gaze remains largely the same, at least at short viewing distances^36^. However, in each case the same qualitative trends were found between stimuli.

Trials were presented using Matlab and the Psychophysics Toolbox extensions^37^,^38^,^39^. In each ‘interval’ the stimulus was displayed at full contrast for 200 ms against a grey background, and was faded on and off with a 100 ms raised cosine ramp on either side of full contrast presentation. After each response there was a 500 ms inter-trial interval, consisting of a blank grey screen, and in the two-interval task there was an additional 500 ms inter-stimulus interval, also consisting of a blank grey screen.

### Procedure

**Figure 2:**
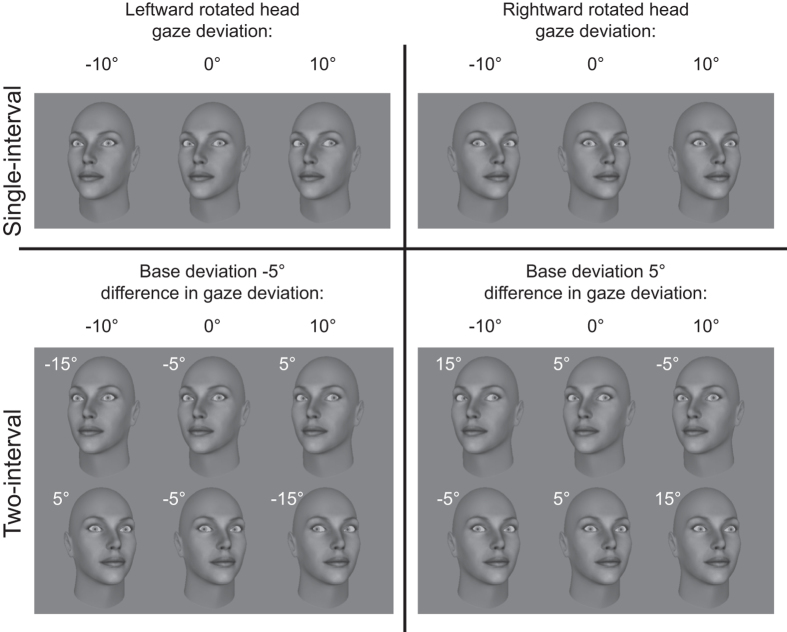
Example stimuli in each task. The top panel shows stimuli used in the single-interval task, where only one face was presented in each trial, either oriented to the left or the right, and with the eyes deviated between −10° and 10°. The bottom panel shows stimuli from the two-interval task, where, on each trial, a (vertical) pair of stimuli would be presented in random order. The deviation of the eyes varied between −10° and 10° from a base orientation of either −5° or 5°, the actual deviation of the eyes is shown in white.

Participants were asked to complete two different tasks, a single-interval task and a two-interval task. Stimuli from each task are shown in Fig. 2. In the single-interval task participants were shown a single face on each trial. The face was rotated 15 degrees either to the left or the right. The method of constant stimuli was used to measure subjectively direct gaze: on each trial the eyes were deviated in one of 11 angular offsets relative to direct (between −10*°* and 10*°*, in steps of 2*°*). The participant was asked to indicate whether the face was looking to the left or the right of them, using the 1 and 2 keys, to indicate left and right respectively. In the two-interval task participants were shown two faces (of the same identity) on each trial; one with the head rotated to the left and one with the head rotated to the right. Again, the method of constant stimuli was employed, this time to measure the mean difference in eye deviation between the two faces at which their gaze appeared equally close to direct (i.e. equally averted in either the same or opposite direction): on each trial the eyes were deviated an equal amount in opposite directions by one of 11 angular offsets (from −10*°* to 10*°*, in steps of 2*°*) relative to a base deviation of +/−5*°*. Participants were asked on each trial to indicate which face was looking more directly at them, using the 1 and 2 keys to indicate the first or second face respectively. In each task, stimuli were presented for 400 ms and participants completed 20 trials per stimulus/stimulus pair, making a total of 440 trials. In total the two tasks took approximately 1 hour to complete and the order in which the tasks were completed was counterbalanced between participants. After one session, 25 participants returned approximately 1 week later to complete another session, in which both tasks were repeated by each participant.

### Analysis

For the single-interval task, the proportion of trials for which the participant indicated that the gaze was offset to the right of them was plotted for each gaze offset, for the left and right rotated heads separately. Logistic functions were fit to these data using the method of least squares. Subjectively direct gaze was taken as the degree of gaze offset at which 50% of trials would be reported as rightward gaze, or the point of subjective equality (PSE). The influence of head rotation on the perceived direction of gaze was taken as half the distance between the PSEs for the leftward- and rightward-rotated heads.

For the two-interval task, the proportion of trials in which the participant indicated that the rightward-rotated head had more direct gaze was plotted for each difference in gaze offset, for the 5° and −5° base offsets separately. Logistic functions were fit to these data using the method of least squares. The PSEs of these functions measure the difference in gaze offset at which the leftward and rightward head’s gaze appeared equally close to direct. The average of these PSEs for the 5° and −5° base offsets was taken as the influence of head rotation on the perceived direction of gaze.

Participants were excluded from further analysis if any of the slopes of the logistic functions fitted to their data were greater than the largest gaze deviation tested, as this indicated that the results might not be reliable. Further inspection of the data revealed that 9 of these participants appeared to be responding to the direction of head orientation instead of gaze direction. The responses of the other 5 observers did not vary markedly with either gaze direction or head orientation.

**Figure 3:**
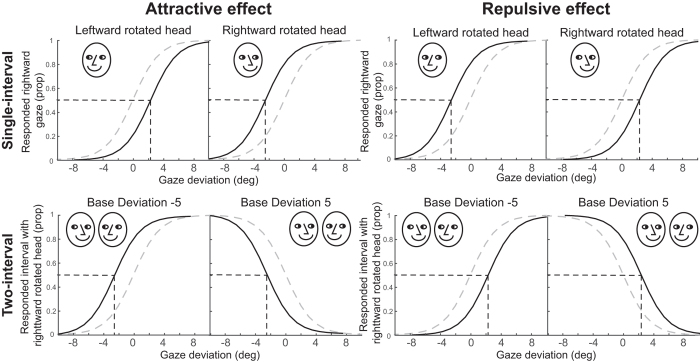
Hypothetical effects of head orientation on PSEs in the single- and two-interval tasks. In all cases the grey dashed line shows a function unaffected by head orientation – the PSE is at 0°, or direct gaze. The top panels show the functions in the single-interval task for attractive and repulsive effects in leftward and rightward rotated heads. The attractive effect causes the PSE to be shifted in the opposite direction of head rotation, indicating responding in the same direction as head rotation over a broader range of gaze deviations. The repulsive effect shifts the PSE in the same direction as head rotation as the observer indicates over a smaller range of gaze offsets that gaze is in the same direction as head rotation. The bottom panels show the functions in the two-interval task for attractive and repulsive effects on base deviations of −5° and 5°. The attractive effect shifts the PSE to the left, as more direct gaze is perceived in the rightward rotated head. The repulsive effect shifts the PSE to the right as more direct gaze is perceived in the leftward rotated head. A bias in the two-interval task for responding to the interval containing the rightward rotated head would shift the psychometric functions for the two base deviations in opposite directions, changing the proportion of responses where the functions intersect but, crucially, leaving the average PSE unaffected.

A positive influence of head rotation on the perceived direction of gaze corresponds to a repulsive effect; gaze direction is perceived as more in the opposite direction of the head orientation. A negative influence of head orientation corresponds to an attractive effect; gaze direction is perceived as more in the same direction as the head is oriented. The influence of head rotation can then be used to calculate the weighting of head orientation on perceived gaze direction in the dual route model (see ref. 16 for details). Importantly, negative weighting of the head shows a repulsive effect, and positive shows an attractive effect. This relationship, and its correspondence with the PSEs in the single interval task, is described in Fig. 3. If an attractive effect of head rotation on perceived gaze direction is observed, then in the single-interval task the PSEs will be shifted in the opposite direction as head rotation: a larger proportion of responses will be made in the same direction as head rotation, and gaze will need to be deviated further in the opposite direction of the head in order to be perceived as direct, or to be judged with equal frequency as left- and rightward gaze. In the two-interval task, the PSEs for each base deviation will be shifted to the left, as the gaze deviation in the rightward oriented head looks more direct. The opposite pattern is seen with a repulsive effect. Note that in the two interval task, if there is a bias for reporting the interval containing the rightward head then the point at which the functions for right- and left-ward base orientations crossed would move up (above 0.5) but would not shift laterally.

## Results

**Figure 4:**
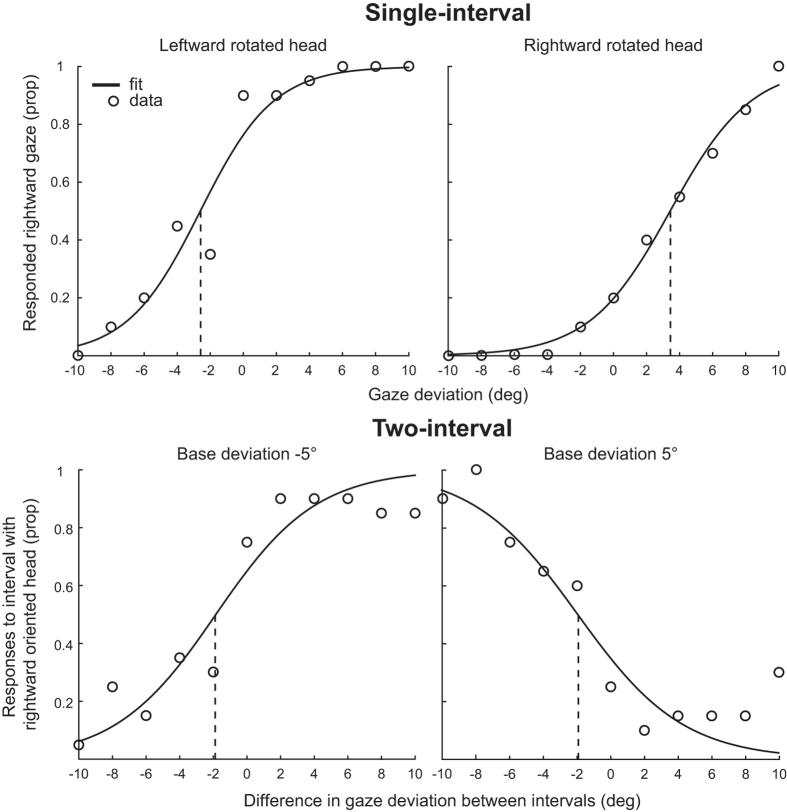
Example of the calculation of PSEs. In the single interval task (top) the proportion of responses indicating rightward gaze for each gaze deviation were fit with a logistic function for the leftward and rightward rotated head separately. In the two interval task (bottom) logistic functions were fit to the proportion of responses for the interval containing the rightward oriented head across each difference in gaze deviation for the two base orientations separately. In all cases the PSE was taken as the mean of the logistic function, or where it was predicted that either response was equally likely.

PSEs were first calculated by fitting a logistic function to each participant’s responses across each task in each session, as shown for one participant’s data from session 1 in Fig. 4. These were then used to calculate the influence of head rotation on perceived gaze direction.

**Figure 5:**
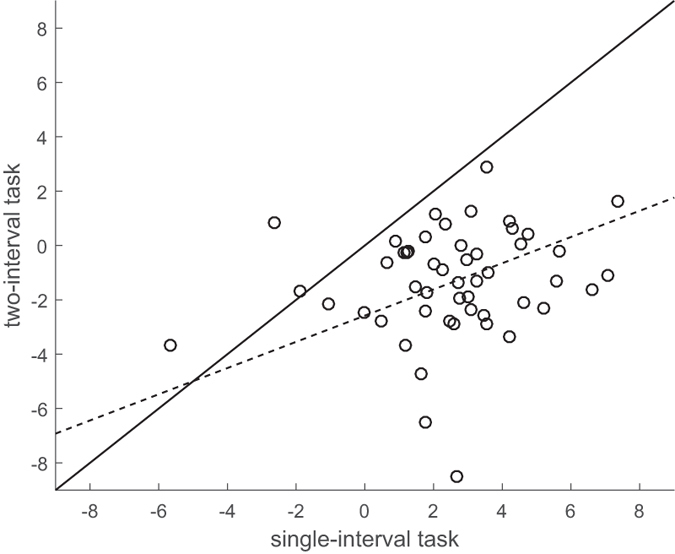
The influence of head orientation on perceived gaze direction. Measures from the single-interval task (x-axis) are plotted against those measured using the two-interval task (y-axis). The solid line is the line x = y, showing the majority of participants showed a stronger effect of head orientation on perceived gaze direction in the single-interval task. The dashed line, y = 0.48x − 2.58, is the best-fit assuming equal within-subject variance in each dimension.

The influence of head rotation on perceived gaze direction measured in the first session was compared across the single-interval and two-interval tasks. A paired samples t-test revealed a significant difference between tasks (*t*(49) = 9.91, *p* < 0.001). A one-sample t-test revealed the influence of head orientation on perceived gaze direction measured in the single-interval task was significantly greater that 0 (*M* = 2.55°; *t*(49) = 7.68, *p* < 0.001) whilst the two-interval task was significantly less than 0 (*M* = −1.35°; *t*(49) = −4.75, *p* < 0.001). To examine the relationship between the tasks, a slope and intercept was fitted to the data by minimising the sum of squared perpendicular distance of each data point to the line, as shown in Fig. 5. The best fitting line (*y* = *0.48x* − *2.58*) revealed a positive correlation between the measures across the two tasks, which was found to be significant by bootstrap resampling with replacement across subjects (*p* < 0.001).

**Figure 6:**
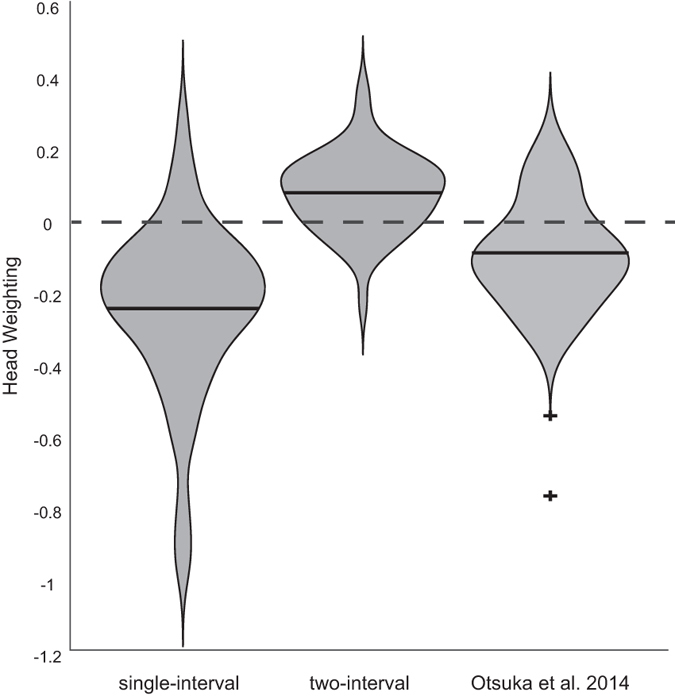
Violin-plots showing the measurement of head weighting from each task compared to the results of Otsuka et al.^15^. The dashed line marks the boundary between a repulsive effect of head orientation (negative head weighting) and an attractive effect (positive head weighting). Solid lines show the means, the width of the plot represents the probability density at each head weighting, and outliers are marked with a plus sign.

These measures were then used to calculate the weighting, w, of head orientation on perceived gaze direction under the dual route model proposed by Otsuka *et al*.^15^ using equation 1:


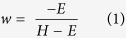


where E is the eye deviation corresponding to the point of subjective equality, and H is the degree of rotation of the head (see ref. 16 for details). Data for each task are shown in Fig. 6 in comparison to the measurements made previously in Otsuka *et al*.^15^. The two-interval task produced results quite different from these previous findings, on average showing a positive head weighting (*M* = 0.07) compared to the negative weighting shown in Otsuka *et al*. (*M* = −0.15) and in the single-interval task (*M* = −0.25).

**Figure 7:**
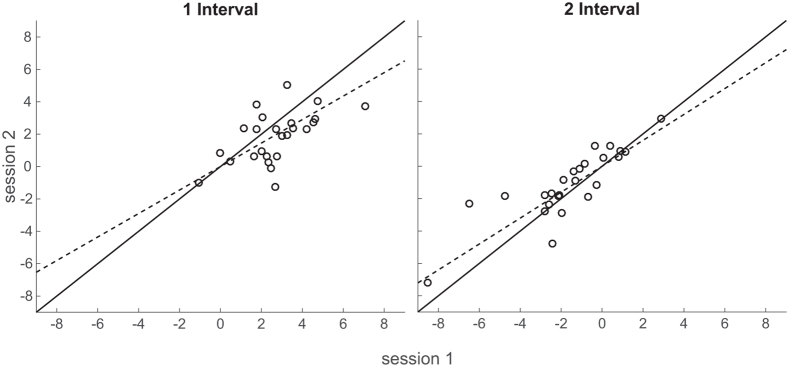
Comparison of measures from sessions one week apart for each task. The measures from the first session (x-axis) are plotted against those from the second session (y-axis). The dashed line shows the best fitting slope to bootstrapped data, whilst the solid line is the line x = y, or a slope of 1, which would indicate perfect replication of measurements from the first session in the second session.

To evaluate the test-retest reliability of each task, the influence of head rotation measured in each session were then compared within each task, for the 25 participants who completed both sessions. A linear slope was fitted to the data (plotted one session against the other, as in Fig. 7) by minimising the sum of squared perpendicular distance of each data point to the line. Positive slopes of 0.73 and 0.80 were found for the single-interval and two-interval tasks, respectively. To test whether the measures differed significantly between sessions, fitted slopes were then compared to a slope of 1, which would signify a perfect replication of measures in the second session. 1000 bootstraps of the data (with replacement) showed slopes significantly different from 1 in the single-interval task (*p* = 0.01), but not in the two-interval task (*p* = 0.07) (although it should be noted that slopes were not significantly different from each other (*p* = 0.32)).

## Discussion

The single- and two-interval tasks produced significantly different measures of the influence of head orientation on perceived gaze direction. The results of the two-interval task suggest the direct attractive effect of head orientation on perceived gaze direction is far stronger than has previously been measured. Whilst observers’ responses in the single-interval task indicated, on average, a negative weighting of head orientation on perceived gaze direction, as in previous experiments using this task design, the two-interval task indicated that this weighting may actually be positive. That is, the orientation of the head has a strong attractive effect on perceived gaze direction that more than counteracts the indirect repulsive effect caused by the altered view of the eyes as the head rotates.

If response bias is the sole difference between the measurements in each task, then the results of this experiment would indicate that observers reliably adopt a large repulsive response bias in the single-interval task that counteracts the direct attractive effect of head orientation. Given the large difference in measurements it is worthwhile questioning whether the only difference between the tasks was the response bias present in the single-interval task. It is unlikely that superficial task differences, such as greater task demands or working memory load in the two-interval task would cause such a difference. There is some evidence that overall performance in the two-interval task was poorer, as many participants made some errors at the extreme gaze deviations tested (proportions of responses to the interval containing the rightward oriented head did not range from 0 to 1 across the range of gaze deviations tested, but often from slightly above 0 to slightly below 1, as demonstrated in Fig. 4). A similar pattern of performance has been demonstrated in previous experiments using an analogous task to measure orientation perception in the tilt illusion^32^. In the current context, the errors could have been the result of the task being more difficult, or because, as the difference in gaze deviation between the two intervals became large, both intervals appeared to clearly contain averted gaze, and thus responding to which was more direct may have been more difficult. Nevertheless, overall drops in performance at the extremes should not cause a systematic shift the PSE (the critical measure here) as they would be expected to occur symmetrically for positive and negative differences in gaze deviation between the two intervals.

Furthermore, had the measures in the two-interval task been affected by erroneous responding caused by increased task demands or working memory load, then we would expect decreased reliability in the measure. On the contrary, in comparing measurements taken one week apart, both the single- and two-interval tasks were shown to provide fairly reliable measures. When measurements across sessions were plotted against each other, the measurements showed a strong correlation, with the linear slopes fitted to the data exceeding 0.7 (Fig. 7). The slopes of the two tasks did not differ significantly, however, the single-interval task did differ significantly from perfect reliability (a slope of 1) whilst the two-interval task did not, suggesting that the two-interval task may even be slightly more reliable.

The large difference between the measurements is also curious because previous studies comparing single- and two-interval designs found no significant differences between tasks^32^. Thus the difference between tasks demonstrated here must be specific to judgements of gaze direction. It remains possible that some difference in the processing of perceptual information for judging gaze direction across the tasks is contributing to the difference in the measured effect of head orientation. For instance, observers may be integrating more information from the head in the two-interval task given that they are comparing the gaze of two oppositely oriented heads, whereas, in the single-interval task, it may be possible to down-weight the head and preferentially process information from the eye region. Or, there could be more pronounced sequential effects of stimulus presentation in the two-interval task given that the stimuli are presented in closer temporal proximity than in the single-interval task. These possibilities are interesting and deserve attention in future research, as they speak to the context specificity with which gaze direction is processed. Indeed, the results support findings that differences in task structure can vary the perceptual effect of head orientation with gaze direction, as has been found when varying the viewing distance of the observer^36^, and presenting stimuli in the periphery^14^. The perceptual effect of head orientation on perceived gaze direction may depend on how the perceptual information can be optimally processed for the task at hand.

Although the summary statistics superficially provide what appears to be a marked difference in the measured influence of head orientation on perceived gaze direction depending on the task employed, the data actually show pronounced inter-individual differences. Whilst on average we observer a repulsive effect of head orientation in the single-interval task and an attractive effect in the two-interval task, some participants showed a repulsive effect in both tasks (12/50 observers), and others, an attractive effect in both tasks (4/50 observers). The examination of the effect of head orientation on perceived gaze direction cannot be conducted in a nominal manner (attractive vs. repulsive effects) but must consider the relative weightings of the direct and indirect effects of head orientation.

When a bias-minimising two-interval task was employed, the direct attractive effect of head orientation on perceived gaze direction was measured to be stronger than previously reported. Further, differences in perceived direction of gaze that accompany the rotation of the head cannot be explained solely by response bias, rather the rotation of the head causes genuine perceptual changes in perceived gaze direction. The two-interval task was shown to be somewhat more reliable than the single-interval task and is recommended for examining the influence of head orientation on perceived gaze direction when the possibility of differences in response bias differ between groups tested, such as in clinical populations. The large differences in measurements between tasks speaks to the context specificity with which head orientation can affect the perceived direction of gaze – there may not be a single way in which information from the head is weighted for the perception of gaze direction, but rather, the weighting of head orientation may be optimised for the processing of the current perceptual information and the task at hand.

## Additional Information

**How to cite this article**: Balsdon, T. and Clifford, C. W. G. A bias-minimising measure of the influence of head orientation on perceived gaze direction. *Sci. Rep.* **7**, 41685; doi: 10.1038/srep41685 (2017).

**Publisher's note:** Springer Nature remains neutral with regard to jurisdictional claims in published maps and institutional affiliations.

## Figures and Tables

**Figure 1:**
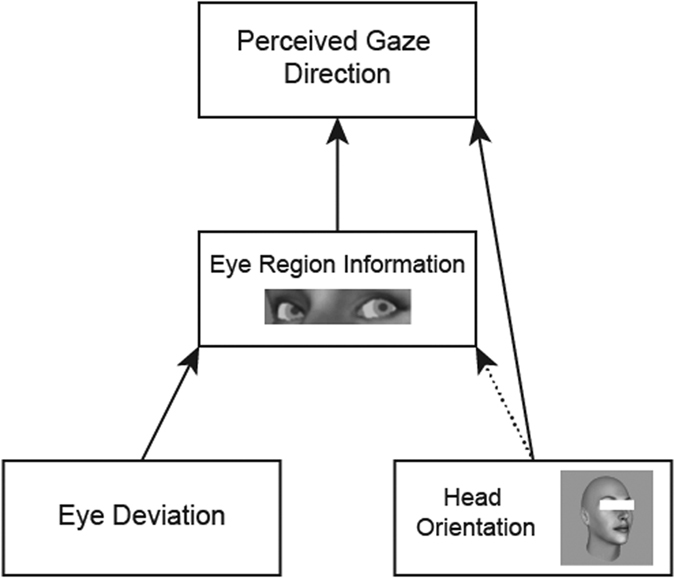
Dual Route Model for the influence of head orientation on perceived gaze direction. Head orientation has an indirect repulsive effect (dotted path) on perceived gaze direction via the changed eye region information; as the head turns the relative amount of visible sclera on one side of the pupil changes. This repulsive effect is somewhat counteracted by the direct attractive effect of head orientation (solid line), which pulls the perceived gaze direction back towards the direction of the head.
